# Severe Intracranial Infection

**DOI:** 10.1016/j.acepjo.2024.100026

**Published:** 2025-01-13

**Authors:** Shinnosuke Fukushima, Takumi Fujimori, Koji Iio, Hideharu Hagiya

**Affiliations:** 1 Department of Infectious Diseases, Okayama University Hospital, Okayama, Japan; 2 Department of Bacteriology, Okayama University Graduate School of Medicine, Dentistry and Pharmaceutical Sciences, Okayama, Japan; 3 Microbiology Division, Clinical Laboratory, Okayama University Hospital, Okayama, Japan

**Keywords:** bloodstream infection, brain abscess, mixed infection

## 1 Case Presentation

A 59-year-old man with a history of alcoholism presented to our hospital with a sudden onset of consciousness disorder. Head contrast-enhanced computed tomography showed multiple subdural abscesses and space-occupying lesions in the maxillary and frontal sinuses (Fig). Blood cultures drawn on the second day detected *Streptococcus constellatus* and Gram-negative bacilli, the latter of which was identified as *Dialister pneumosintes* by matrix-assisted laser desorption/ionization time-of-flight mass spectrometry (MALDI Biotyper; Bruker Daltonics) with an identification score of 2.29. A 16S ribosomal RNA gene analysis using the Basic Local Alignment Search Tool demonstrated a high concordance rate of 99.91% with the reference strain (GenBank accession number: LC037225.1). The patient underwent surgical drainage of the brain abscesses and was consequently transferred to another hospital.

**Figure:**
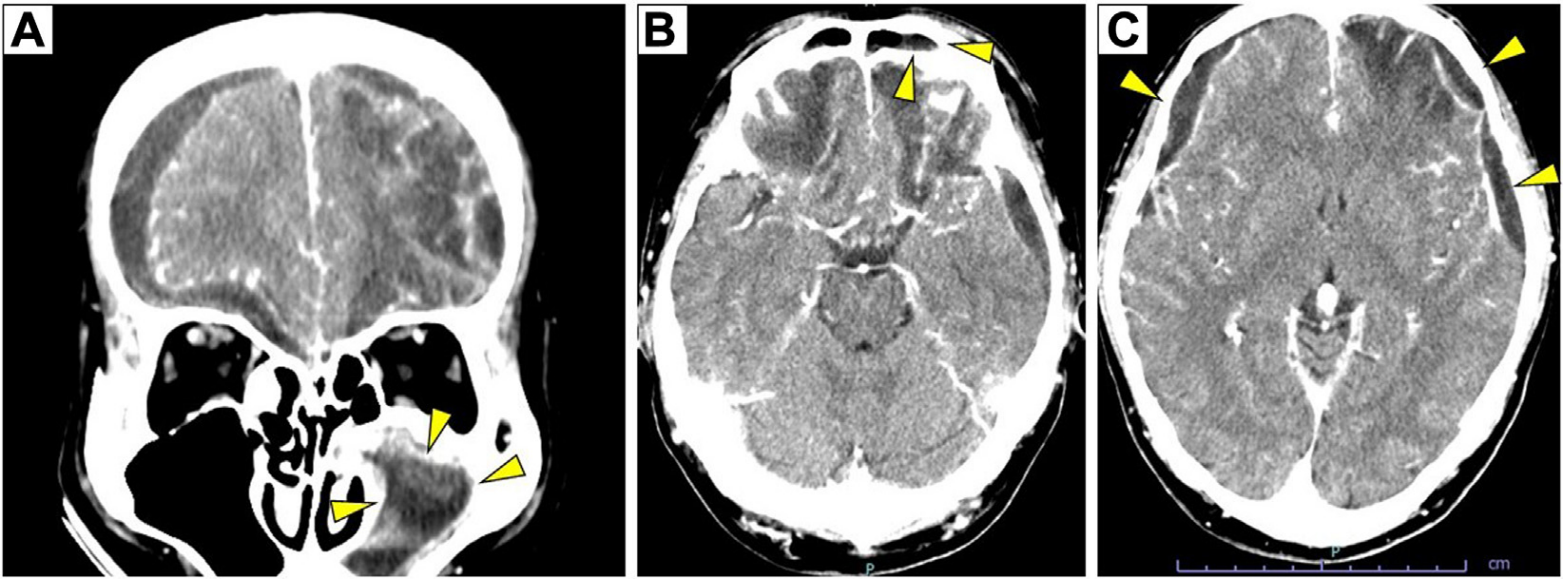
Contrast-enhanced computed tomography of the head. Contrast-enhanced computed tomography imaging demonstrates left-sided maxillary and frontal sinusitis (A, B), with evidence of multiple subdural abscess formations (C).

## 2 Diagnosis: Severe Intracranial Infection Caused by *Dialister Pneumosintes*

*D pneumosintes* is an anaerobic or microaerophilic Gram-negative coccobacillus that often requires 16S ribosomal RNA gene sequencing for accurate identification.^1^^,^^2^ *D* *pneumosintes* is rarely detected in blood cultures and has been reported to cause head and neck infections such as brain abscesses and Lemierre’s syndrome.^2^, ^3^, ^4^ A previous case of brain abscesses was diagnosed as a mixed infection involving *D* *pneumosintes* and *Streptococcus anginosus.*^3^ In the present case, polybacteremia mixed with *D pneumosintes* was observed, and the brain abscesses were suspected to have originated from the sinuses. Our case highlights the pathogenicity of *D pneumosintes* in brain abscess formation.

## Funding and Support

By *JACEP Open* policy, all authors are required to disclose any and all commercial, financial, and other relationships in any way related to the subject of this article as per ICMJE conflict of interest guidelines (see www.icmje.org). The authors have stated that no such relationships exist.

## Data Availability

The datasets used during the current study are available from the corresponding author on reasonable request.

## Conflict of Interest

All authors have affirmed they have no conflicts of interest to declare.
